# Low to Moderate Prenatal Alcohol Exposure and Neurodevelopmental Outcomes: A Narrative Review and Methodological Considerations

**DOI:** 10.35946/arcr.v43.1.01

**Published:** 2023-03-16

**Authors:** Gretchen Bandoli, Shana Hayes, Erin Delker

**Affiliations:** Department of Pediatrics, University of California San Diego, La Jolla, California

**Keywords:** alcohol, prenatal alcohol exposure, fetal alcohol spectrum disorders, epidemiology, low, light, or moderate exposure, neurodevelopment

## Abstract

**PURPOSE:**

Although abstinence is recommended in pregnancy, many pregnancies are exposed to alcohol. Observational studies of the effects of low to moderate prenatal alcohol exposure (PAE) and neurodevelopmental outcomes have yielded inconsistent results, with some studies finding an increased risk of adverse neurobehavioral and cognitive outcomes, and other studies finding no changes or reduced risk of the same outcomes. The purpose of this narrative review is to summarize these inconsistencies and apply a methodological framework to discuss how different parameters contribute to the findings. The authors also provide recommendations on how to advance future research in this area.

**SEARCH METHODS:**

The PubMed, Web of Science, and Embase databases were searched, along with reference lists of selected systematic reviews and meta-analyses. Search terms used were (infant or child or children or adolescent or offspring) AND (low or light or mild or moderate or low-to-moderate) AND (drinking or alcohol or drinks) AND (pregnancy or prenatal or fetal) AND (neurodevelopment or behavioral or psychological or cognitive or developmental) NOT (mice or rat or fish or animal) NOT (meta-analysis or review). Peer-reviewed original research studies were included if they analyzed associations between an exposure defined and characterized as low/light or moderate PAE with offspring neurodevelopmental outcomes. Animal studies, studies that did not provide clear cutoff points to classify PAE categories, studies lacking an abstinence control group, and studies that did not present a multivariable-adjusted measure of association were excluded.

**SEARCH RESULTS:**

The searches identified 2,422 papers, with 36 papers meeting eligibility criteria. These studies were carried out across nine countries and included samples ranging from approximately 500 to 40,000 participants. Cognitive, academic, socioemotional, and behavioral outcomes were assessed from infancy through age 19.

**DISCUSSION AND CONCLUSION:**

When the findings from the selected articles were summarized by geographic region, exposure definition, or neurodevelopmental outcome, no consistent observations or patterns emerged between low to moderate PAE and offspring outcomes. Although some studies found positive (i.e., beneficial) associations between low to moderate PAE and outcomes (primarily outcomes related to cognition) and others found negative (i.e., detrimental) associations (primarily for behavioral outcomes), most findings were null (i.e., showed no effect of PAE). The heterogeneity in study results is likely due to methodological issues, including residual confounding, effect measure modification, and exposure misclassification that make synthesis of studies difficult. Alternative study designs, including longitudinal trajectory analysis, sibling design, negative controls, and instrumental variable analyses, may reduce biases and are discussed. To date, the consequences of light to moderate levels of PAE on neurodevelopment remain unresolved; studies that advance methodological rigor will be important contributions to the field.

Prenatal alcohol exposure (PAE) is a necessary cause of fetal alcohol spectrum disorders (FASD), a group of alcohol-related conditions characterized by neurodevelopmental problems. Although PAE is associated with many adverse physical, neurodevelopmental, and social outcomes, the most commonly studied are neurodevelopmental—primarily behavioral and cognitive—outcomes. Associations between heavy PAE (which is inconsistently defined) or binge PAE (defined as consuming four or more drinks in about 2 hours in women, or the amount of alcohol necessary to achieve a blood alcohol concentration of 0.08% or higher^1^) and adverse neurodevelopmental outcomes have been well documented in the literature.^2^–^4^ However, findings regarding associations between lower levels of PAE and neurodevelopmental outcomes are inconsistent, with summations to date yielding, at best, inconclusive results.^5^ Moreover, there is no consensus in the literature on the definition of “low to moderate” PAE—or, correspondingly, the level of harm that low to moderate PAE may cause^5^—leaving pregnant individuals and their clinicians ill-equipped to assess risk of exposure.

Several systematic reviews^6^–^10^ and at least four meta-analyses^2^,^11^–^13^ have assessed associations between low to moderate PAE and child neurodevelopmental outcomes. Pooling results from studies published through 2012, Flak and colleagues reported a small positive association between mild to moderate PAE (defined as up to six drinks per week) and child cognition (beta estimate 0.04; 95% confidence interval *CI* [0.00, 0.08]); seven studies).^2^ They also identified a modest association between moderate PAE (defined as up to six drinks per week, including some individuals who consumed at least three drinks per week) and adverse behavioral outcomes, such as problems with behavior regulation and increased demand for attention at ages 9 months to 5 years (beta estimate −0.15; 95% *CI* [−0.28, −0.03]; three studies).^2^ A more recent meta-analysis, pooling studies published through 2020, also found that low to moderate PAE (author characterized, or one to fewer than seven drinks per week) was associated with adverse behavioral outcomes (i.e., attention problems) at ages 6 to 17 (*OR* 1.21; 95% *CI* [0.88, 1.65]; six studies). However, the magnitude of associations estimated varied dramatically across studies.^11^ Dissimilar to the prior studies, a meta-analysis specifically examining the effect of low to moderate PAE (≤ 20 g/week to ≤ 50 g/week) on risk of attention-deficit/hyperactivity disorder (ADHD) reported no effect (*OR* 0.96; 95% *CI* [0.86, 1.02]; six studies).^12^ Studies included in this review used a few different measures to assess ADHD symptoms between ages 3 and 14.^12^

The impetus remains to better understand the relationship between low to moderate PAE and offspring neurodevelopmental outcome. Although most authoritative bodies recommend complete abstinence from alcohol during pregnancy, PAE continues to be common, particularly in the early weeks of gestation prior to pregnancy recognition. In surveys conducted by the Behavioral Risk Factor Surveillance System between 2018 and 2020 in the United States, about 14% of pregnant women reported past 30-day alcohol use.^14^ It is possible that the inconclusiveness in previous research findings is not driven by a paucity of research, but by inconsistencies in methodology used across studies. The purpose of this narrative review is thus threefold. First, it briefly summarizes select literature of low to moderate PAE and neurodevelopmental outcomes, noting consistencies and inconsistencies in findings. Second, it reviews methodological issues that limit valid ascertainment of the effects of low to moderate PAE on offspring neurodevelopmental outcomes. Third, it discusses alternative study designs that may address key methodological issues for consideration in future research.

## Methods

### Search Strategy

The PubMed, Embase, and Web of Science databases were searched on June 13, 2022. The search terms used to identify articles were (infant or child or children or adolescent or offspring) AND (low or light or mild or moderate or low-to-moderate) AND (drinking or alcohol or drinks) AND (pregnancy or prenatal or fetal) AND (neurodevelopment or behavioral or psychological or cognitive or developmental) NOT (mice or rat or fish or animal) NOT (meta-analysis or review). Results of the search strategy were checked against reference lists of existing systematic reviews and meta-analyses to verify that the search was comprehensive.^2^,^6^,^11^,^12^

### Eligibility Criteria

The inclusion criteria for this review were: (1) peer-reviewed original research study; (2) human participants; (3) PAE characterized as low, light, mild, moderate, or low to moderate; and (4) any neurobehavioral or developmental outcome in offspring. Exclusion criteria were: (1) reference group other than “no PAE” or abstinence; (2) no parameters provided for the quantity or frequency of alcohol exposure that was used to classify individuals as having low, light, mild, moderate, or low to moderate PAE; (3) inability to separate PAE from co-occurrence with exposure to other substances; (4) no adjustment for confounding variables; (5) no measure of association presented for low/light or mild/moderate PAE categories (e.g., exposure was analyzed as a continuous variable); (6) quasi-experimental study design; and (7) alcohol exposure not specific to the pregnancy period.

### Data Abstraction and Synthesis of Results

One of two reviewers examined titles and abstracts of each article to identify articles meeting criteria for full text review. During full text review, one of two reviewers abstracted the following information from each study: (1) author and year; (2) data source and sample size; (3) study setting and offspring birth years; (4) definition of low/light PAE; (5) definition of mild/moderate PAE; (6) definition of one drink or one unit of alcohol; (7) timing of PAE measurement; (8) outcome, outcome measurement tool, and age of offspring at outcome measurement; (9) confounding variables considered; and (10) results. These data are summarized in Appendix 1.

## Results

**Figure 1:**
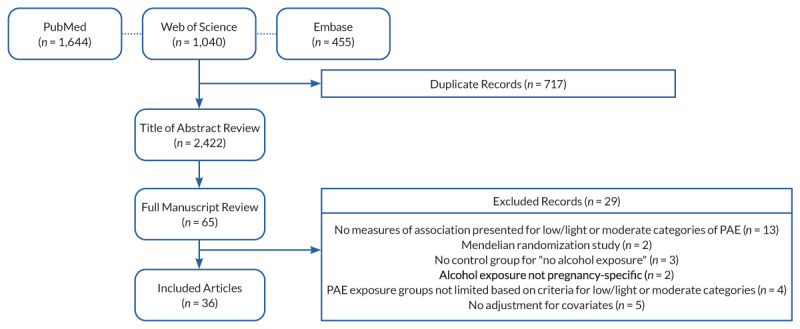
Sample selection for inclusion in this review. *Note:* PAE, prenatal alcohol exposure.

The search of PubMed (*n* = 1,644), Embase (*n* = 455), and Web of Science (*n* = 1,040) databases yielded 2,422 unique records, 65 of which met criteria for full text review. Of these, 29 were excluded (see Figure 1 for details of exclusions), and 36 were included in the final set. With a few exceptions, most studies reported no associations between low or moderate PAE and offspring neurodevelopment. Appendix 1 synthesizes the results by study location, exposure definition, timing of exposure measurement, and neurodevelopmental outcome.

### Study Location

Included studies reported data from Australia (eight studies);^15^–^22^ New Zealand, Australia, Ireland, and the United Kingdom (one study);^23^ Denmark (nine studies);^24^–^32^ Denmark and Finland (one study);^33^ Japan (one study);^34^ South Africa (one study);^35^ the United Kingdom (nine studies);^36^–^43^ and the United States (six studies).^44^–^49^ Of note, many of the studies published from the same country used data from the same source. For example, half of the Australian studies used the Western Australian Health Survey data;^15^,^21^seven of the nine Danish studies used data from the Lifestyle During Pregnancy Study (based on a sample from the Danish National Birth Cohort);^26^–^32^ and six of the nine U.K. studies used data from the Millennium Cohort Study.^36^–^39^,^41^,^50^Individual studies with the same data source often used the same definition of PAE but varied by study outcome or offspring age at neurodevelopmental assessment.

Some patterns were observed in study findings by country. For example, all studies from Denmark and Denmark/Finland reported mostly no effect of low to moderate PAE (i.e., null findings). Two studies—one using data from the Danish National Birth Cohort^25^ and one using data from the Aarhus Birth Cohort^24^—published findings suggesting potential protective associations between low PAE and ADHD in both sexes^24^ and between low to moderate PAE and internalizing problems among boys.^25^ Results from each of the Australian studies were also mostly null, although two studies reported some evidence for worse behavioral problems with low PAE,^15^,^17^ and three studies reported some evidence for better behavioral,^16^ cognitive,^18^ and academic outcomes with low PAE.^21^ Studies from the United Kingdom focused on potential sex differences in the associations between low to moderate PAE and neurodevelopmental outcomes. Two studies using data from the Avon Longitudinal Study of Parents and Children reported worse behavioral rating scores among girls (but not boys) exposed to light PAE compared to children without PAE.^40^ Two analyses using data from the Millennium Cohort Study reported that light PAE was associated with better cognitive scores^36^,^37^ and lower behavioral scores^37^ among boys relative to boys without PAE. Of the six studies from the United States, findings from three studies suggested worse behavioral outcomes with light to moderate PAE compared with no PAE,^45^,^46^,^48^one study noted a possible protective association between light PAE and autism spectrum disorder (ASD),^44^ and the remaining two studies showed null results^47^,^49^ (see Appendix 1).

### Prenatal Alcohol Exposure

There was tremendous heterogeneity in how low/light or moderate PAE were defined. Most studies included in this review defined these categories based on the average quantity of drinks consumed per week. Of these, low/light PAE was most frequently defined as averaging one to four units of alcohol per week, with specific definitions ranging from less than one unit per week to less than seven units per week. The studies using the higher threshold of fewer than seven units per week were mostly conducted in Australia, matching the definition of low-risk drinking for women.^15^–^17^,^20^,^21^,^52^ Many studies also considered units per occasion in their drinking definition. For example, most studies using data from the Millennium Cohort study defined low PAE as “not more than one to two units per week or peroccasion;”^36^–^38^,^50^ and most studies using the Western Australian Health study defined low PAE as “not more than seven drinks per week and up to two drinks per occasion.”^15^,^19^,^20^,^21^,^52^ The study from South Africa considered only the number of drinks per occasion (mild to moderate: not more than three drinks in one sitting, never binge drinking),^35^ and the study from Japan considered only frequency of drinking (low PAE: drinking rarely to one to four times per month).^34^ For moderate PAE, most definitions were between three and 10 drinks per week. Some considered drinks per occasion in addition to drinks per week (range from more than two to five drinks per occasion).

The definition of a drink or unit of alcohol also varied by country. A standard drink was typically defined as 10 grams of pure alcohol in Australia; 12 grams in Denmark; and 8 grams or half a pint of beer, a glass of wine, or a single measure of spirits or liquor in the United Kingdom (see Appendix 1). No clear pattern of study findings emerged by definition of low/light PAE, moderate PAE, or unit of alcohol.

### Timing of PAE Assessment

In all studies analyzed, PAE was assessed using self-report by the women as there are no biomarkers that could provide details on the dose and timing of low to moderate PAE. Twenty-two of the included studies collected self-report information on PAE during pregnancy,^16^–^18^,^20^,^22^–^33^,^35^,^40^,^42^,^43^,^47^,^49^ and 14 studies relied on reports obtained after pregnancy,^15^,^19^,^21^,^34^,^36^,^37^,^39^,^41^,^44^–^46^,^48^,^50^ including 10 studies with reporting occurring within 1 year of delivery.^15^,^19^,^21^,^36^–^39^,^41^,^45^,^50^ Only three studies assessed PAE at the same time as or after the infant outcome was assessed (i.e., retrospectively). Of these, two reported that light to moderate PAE was associated with worse behavioral problems at age 23 months^48^ and at ages 9 to 10 years;^46^ and the third—a case control study examining associations with ASD—reported mostly null findings, with the exception of a protective association between one to two drinks in the first month of pregnancy and lower odds of ASD.^44^

### Neurodevelopmental Outcomes

The studies included in this review assessed neurocognitive, academic, and behavioral outcomes when offspring were between the ages of 9 months and 19 years. None of the included studies reported worse neurocognitive outcomes with low to moderate PAE. For example, studies examining associations between PAE and Bayley Scales of Infant Development mental development and psychomotor indices in infants ages 12 to 24 months reported mostly null findings,^17^,^20^,^42^,^52^ with one study suggesting that low PAE in the second and third trimesters was associated with better cognitive outcomes.^18^ Three studies examining associations between PAE and IQ at about age 5 also reported null findings.^23^,^26^,^29^ Four studies using data from the Millennium Cohort Study that assessed cognitive development with the British Abilities Scale reported protective associations between light PAE and cognitive outcomes in boys at age 3^36^ and age 5,^37^ but null associations when the boys were evaluated at ages 7 and 11.^38^,^39^

For measures of socioemotional and behavioral health, most studies used data ascertained with either the Strength and Difficulties Questionnaire (10 studies^25^,^30^,^33^,^36^–^41^,^43^) or the Child Behavior Checklist (CBCL; seven studies^15^,^16^,^22^,^23^,^34^,^35^,^46^). For these measures, offspring age at the time of assessment varied greatly and results were mixed with no clear pattern identified. Others studies examined PAE in relation to specific diagnoses, including ADHD (two studies)^24^,^41^ and ASD (two studies),^44^,^50^ with all studies reporting null or protective results. Three studies used infant behavior rating checklists to measure behavioral outcomes in infants ages 9 to 24 months. All three studies reported that light to moderate PAE was associated with worse behavioral outcomes in infancy, including increased infant difficultness,^48^ poorer social engagement,^45^ and more sensation seeking.^17^

Regarding academic outcomes, three studies reported null associations between PAE and academic achievement.^39^,^40^,^49^ One study reported that low PAE was associated with lower odds of meeting Australian numeracy academic benchmarks, but had no effect on meeting reading, spelling, or writing benchmarks.^21^

## Discussion and Comment

Despite a large and growing evidence base, making inferences about the effects of light to moderate PAE on neurodevelopmental outcomes in offspring remains difficult. Heterogeneity in effect estimates has persisted over the past 20 years, with both harmful and protective effects observed. Due to differences in study approach (summarized in Appendix 1) and methodological limitations, the diverse study results are difficult to synthesize. The following section further details these issues.

### Methodological Issues With the Study of Light to Moderate PAE

#### Definition of exposure

Currently, there are no standard criteria or consensus for defining low/light or moderate levels of PAE. This heterogeneity in exposure definitions has undoubtedly contributed to inconsistencies of effect estimates and has limited the ability to make comparisons between studies. Even in abstracting data for this review, the authors were limited to papers that self-classified exposure into low/light or moderate PAE. Undoubtedly, many additional studies have investigated similar exposure levels but did not label them as such and therefore could not be included in this review because they were not identified in the literature searches.

#### Nondifferential exposure misclassification: Static measurement of PAE

Studies of PAE often use time-insensitive, or static, categorizations of exposure (e.g., categorizing the entire pregnancy as “high” consumption based only on PAE at conception) that fail to incorporate the dynamic changes in exposure that occur across pregnancy. These changes typically occur around the time of pregnancy recognition (which can be highly variable across individuals) when many women reduce their consumption or abstain from alcohol. For some pregnancies, changes also occur later in pregnancy as the perceived “risk period” for fetal development passes and women feel more comfortable resuming some level of alcohol use. The timing of these transition points may be informative with respect to offspring development but frequently is not examined. Further, when time-varying exposures are collapsed into static variables, a resulting exposure misclassification may lead to attenuation of effect estimates. This is particularly of concern when studying the consequences of low and moderate PAE, where modest effect estimates can disappear entirely due to exposure misclassification.

#### Differential exposure misclassification: Recall bias

There are no validated biomarkers that reflect prenatal exposure several years after birth; consequently, PAE is frequently measured by maternal recall. The gold standard approach in the collection of PAE information is through timeline follow-back^53^ during pregnancy before the outcome is known, after which recall bias may occur. Due to stigma, individuals who perceive neurobehavioral problems in their offspring may be more likely to underreport their prenatal alcohol consumption relative to individuals who perceive no problems, which in cases of extreme underreporting could result in estimated protective effects of PAE. Although it is much more feasible to collect information retrospectively once children are at suitable ages for neurodevelopmental evaluation, the impact of this approach on internal validity must be critically evaluated.

However, when assessing studies that rely on recalled PAE, research has noted that recalled alcohol exposure is strongly predictive of pregnancy, dysmorphic, and neurodevelopmental outcomes.^54^ Further, validation studies comparing prospective and retrospective reports found that retrospective reports of maternal drinking reflect higher levels of consumption than prospective reports obtained during the prenatal period.^55^–^58^ Thus, although collection of consumption data during pregnancy remains preferable, retrospective information should not be discounted simply due to potential recall bias, and actually may be more accurate in some groups, particularly those who perceive stigma when reporting during pregnancy. Notably, however, the ability to recall PAE after pregnancy may differ by level of prenatal alcohol use. Women who did not consume any alcohol as well as those who habitually had high levels of consumption both may have more accurate recall than women who infrequently consumed alcohol, particularly with respect to the precise timing and amount of alcohol. This differential exposure misclassification could move estimates of PAE effects in either direction (toward or away from the null).^59^

#### Confounding variables

To validly estimate a causal effect of PAE on neurodevelopment, the exposure groups must be interchangeable. This means that variables that could confound the association between light to moderate PAE and offspring outcomes are equally balanced between alcohol-exposed and non–alcohol-exposed offspring. In observational studies, many socioeconomic and psychosocial factors have been associated with alcohol consumption patterns. A study of more than 6,000 women in Australia examined maternal factors associated with patterns of alcohol consumption before, during, and after pregnancy. The analysis found that compared to women with light prenatal alcohol consumption (0.4 drinks per day pre-pregnancy, early pregnancy cessation), women with high levels of alcohol consumption (2.5 drinks per day pre-pregnancy, 0.6 drinks per day during pregnancy) were more likely to have a lower income, be single or divorced, be pregnant for the first time, not attend church, report depression or anxiety, have high maternal adversity, and have adverse health-related lifestyle behaviors (e.g., smoking, little exercise, poor sleep).^60^ Within the same study, women who abstained from alcohol pre-pregnancy through postpartum were also more likely to have lower income, have more children, and have adverse health-related lifestyle behaviors compared to women with light prenatal consumption.^60^ Similarly, in a study of 4,000 pregnant women in the United Kingdom, women of higher socioeconomic status were more likely to drink wine, which was more likely to be consumed in low to moderate amounts, and less likely to binge drink than those with lower socioeconomic status. Further, being older, being better educated, having a higher social class, being employed, and having a better educated, employed partner were associated with consumption of wine, whereas smoking, lower education, and worse mental health were more strongly associated with consumption of beer.^61^ Other factors that favor positive neurobehavioral outcomes in the offspring were disproportionally shared by women who consumed low to moderate amounts of alcohol. These factors include better diets, earlier use of prenatal vitamins, lower prevalence of mental illnesses such as depression or anxiety, and lower likelihood to use other substances (e.g., marijuana) during pregnancy.^62^ These studies highlight the imbalance in protective factors that often accompany low to moderate PAE that may bias findings, resulting in null or even protective effects when compared to abstinence.

Although these and other factors associated with low to moderate prenatal alcohol consumption that reduce the likelihood of adverse neurodevelopmental outcomes are routinely included in multivariable adjustment, they may still result in unmeasured confounding. Additionally, sample sizes typically limit the ability to include the multitude of variables necessary to establish true exchangeability. As a result (and common in all observational studies), the degree to which residual confounding persists—as evidenced by positive associations often reported between low to moderate PAE and offspring neurodevelopmental outcomes—must be considered because there is no conceivable benefit on these outcomes from PAE itself.^63^,^64^

#### Effect modification

In addition to confounding, which affects the internal validity (bias) of an estimate, the effect modification of the estimate by factors associated with the outcome (which can be termed modifiers or moderators) must be considered. Although not a source of bias, this occurs when the magnitude of the effect varies across levels of a third variable, which may contribute to heterogeneity in effect estimates across studies. Here, the authors hypothesize higher socioeconomic status associated with low to moderate PAE to be the third variable. For example, women consuming low to moderate levels of alcohol in pregnancy, which as previously noted is associated with higher socioeconomic status, may have more resources at their disposal postnatally compared to either women who abstain or women who consume large quantities of alcohol, providing a more enriched environment for the offspring. These factors include the likelihood to breastfeed, high-quality childcare, reduced environmental exposures, higher levels of social support, access to health care, reduced caregiver stress, and educational resources available to the child, which benefit neurodevelopmental outcomes. When effect estimates for PAE are not stratified by these potential modifiers, outcomes most vulnerable to PAE may be masked by the preponderance of protective factors associated with low to moderate PAE.

In summary, the null or protective effects attributed to low to moderate PAE on neurodevelopmental outcomes are likely, at least in part, attributable to unmeasured confounding associated with the exposure as well as to effect modification by postnatal factors that favor children with low to moderate PAE. Many statistical tools exist to address confounding (e.g., propensity score adjustment, inverse probability of treatment weights) and effect modification (e.g., stratification, re-weighting to a standardized sample). However, these options are imperfect because of the strong psychosocial patterning of PAE and insufficient resources to validly measure all potential confounding variables or examine associations across population subgroups. Further, as previously noted, there is a high degree of nuance in the operationalization of PAE, highly likely leading to misclassification of exposure and attenuation of results. To overcome these challenges, researchers have begun applying novel study designs and exposure models to this research. The following section highlights a few such approaches.

### Alternate Study Designs to Address Methodological Issues

The study designs and methodologies discussed in this section—which are by no means exhaustive—specifically target some of the aforementioned threats to validity. Thus, exposure misclassification can be addressed by determining longitudinal trajectories of PAE, and confounding can be limited through use of instrumental variables, sibling designs, and negative control studies. These methods can be used alone or in combination to enhance estimation of causal effects.

#### Longitudinal trajectories of PAE

Efforts to better capture and operationalize the three parameters of alcohol use (timing, dose, and duration) have been ongoing for many years. Initially, researchers manually clustered individuals based upon these characteristics. In one study, investigators created a composite measure of PAE that incorporated dose, pattern, and timing of consumption into a descriptive, categorical variable (e.g., low, moderate, binge drinking less than once per week; binge drinking once or twice per week; high PAE).^15^ When they compared outcomes using these classifications to traditional analytic methods (i.e., average quantity per trimester, average daily exposure across pregnancy, average weekly exposure across gestation), the researchers detected increased odds of anxiety or depression for children with moderate PAE in the composite models that were not evident in the traditional analyses.^15^ Lately, unsupervised machine learning techniques have been incorporated into analyses to identify patterns of use across gestation.

Although several methodologies exist, the underlying premise of longitudinal exposure modeling is to create groups with similar longitudinal exposure patterns to minimize heterogeneity within the assigned trajectory group and maximize heterogeneity between trajectories. In a study employing PAE trajectories in Ukraine, sustained alcohol exposure, even at relatively low levels (about one drink per day), was associated with modest reductions in neurodevelopmental performance at 6 and 12 months of age compared with trajectories of higher PAE with alcohol reduction or cessation earlier in pregnancy.^65^ In the Safe Passage Study (South Africa and the United States), the sustaining trajectory (modeled as maximum drinks per drinking day) was associated with sudden infant death syndrome, whereas the trajectories with similar early pregnancy consumption but earlier cessation were not.^66^ By modeling trajectories, researchers can disaggregate patterns of consumption, even among individuals with low to moderate levels of consumption, to better identify and understand the nuanced risk of adverse offspring outcomes that are often lost when exposure variables are operationalized as static measures.

#### Instrumental variables

Instrumental variable (IV) analysis is another study design that could improve research on low to moderate PAE and address issues of exchangeability. An IV affects the outcome only through its effect on the exposure and is unrelated to potential confounders of the exposure–outcome association.^67^ In experimental studies, the treatment assignment is the IV. However, when experimental studies are not feasible, researchers may use a “quasi-experimental” approach to an IV through “Mendelian randomization,” involving the use of genetic variants that influence the exposure but are unrelated to factors that confound the exposure-outcome relationship.^68^ Three recent studies using data from the Avon Longitudinal Study of Parents and Children (ALSPAC) utilized this design.^61^,^62^,^68^ In onestudy, investigators used analysis of a maternal genetic variant in the alcohol dehydrogenase gene *ADH1B* as an instrument for assessing PAE.^61^ Individuals who carry the rare variant rs1229984 in *ADH1B* have greatly increased enzymatic activity in the oxidation of ethanol to acetaldehyde. This increased activity results in a faster reduction of blood alcohol levels and sharper rise of acetaldehyde in blood and organs, leading to symptoms such as increased heart rate and nausea. Individuals with this variant consume less alcohol, and correspondingly, the fetuses of mothers with the variant have lower PAE. These observations were born out in the ALSPAC data, making this a suitable IV candidate for low to moderate PAE.^61^,^62^,^68^ When the researchers used a traditional analysis of the effects of any alcohol exposure, estimates were largely null, indicating no effect. In contrast, when they stratified the analysis by type of exposure (i.e., preferred type of beverage), they noted positive effects between wine consumption and offspring academic achievement, and negative effects between beer consumption and offspring academic achievement. Given that there should be no difference between the effects of beer and wine when both were converted into standard, equivalent doses, the researchers suggested that the different outcomes were due to the strong social gradient associated with choice of beverage. *ADH1B*, however, was unrelated to potential confounders, but was predictive of alcohol use in pregnancy and, by extension, alcohol exposure to the developing fetus. When authors conducted the IV analysis with *ADH1B*, they found negative effects between PAE and academic achievement at all ages analyzed (i.e., ages 7 to 16).^61^ Similar results were obtained when repeating this analysis with cognitive and educational performance at age 8.^68^

The third study that utilized an IV analysis constructed the IV from the child’s genotype (variants of the alcohol dehydrogenase enzyme). It was hypothesized that offspring alleles, which result in “fast” metabolism of ethanol, would protect against abnormal brain development in infants.^62^ Among offspring born to women with moderate alcohol consumption (one to six units per week during pregnancy), negative associations existed between the presence of genetic variants associated with slow alcohol metabolism and IQ at age 8. These associations were not seen in children with no PAE.^62^ Assuming these findings can be replicated, they are a promising avenue to control for confounding factors while estimating the effects of low to moderate PAE on child health. Researchers should continue to explore additional IVs, specifically searching for stronger IVs than the alcohol metabolism variants, which can be exploited in this framework.

#### Sibling controls

A third promising approach that also addresses issues of exchangeability is a sibling design. In their simplest form, sibling designs compare outcomes among exposure-discordant siblings, which accounts for many shared environmental and familial confounders that are exceedingly difficult to fully adjust for through multivariable analysis. Sibling studies often result in attenuated effect estimates between prenatal exposures and offspring neurodevelopmental outcomes compared to traditional study designs,^69^ highlighting the challenges of residual confounding.

At least three studies have utilized sibling designs to control for shared genetic and environmental confounders when studying PAE.^34^,^70^,^71^ In the first sibling study conducted on approximately 4,000 mother-sibling triads, researchers found that employing the sibling design attenuated the initial multivariable-adjusted results for PAE and attention/impulsivity problems in offspring. However, an association still existed between heavy PAE (≥ 5 days/week) and offspring conduct problems at ages 4 to 11.^70^ In a second study, conducted with 15,000 mother-sibling triads in Norway, researchers detected no effects of low levels of PAE on offspring behavioral problems, and attenuated yet modest effects of “hazardous” PAE on behavioral problems at age 3 when accounting for siblings.^71^ A third study, which included 1,600 sibling pairs in Japan, found that low PAE (drinking one to four times per month or rarely) was associated with greater anxiety problems and internalizing problems. In that study, effect estimates were magnified in the siblings analysis compared to initial multivariable-adjusted results.^34^ Although researchers must consider exposure discordance and factors that changed between births (e.g., birth order, maternal age, socioeconomic factors) that may bias results, this model is a promising strategy to mitigate the persistent problem of residual confounding.

#### Negative controls

Finally, another option that does not require identifying an IV or observing siblings is to employ a negative control design. Such designs compare the effects of PAE on offspring outcome with the effects of similar exposure with no biological relevance to the offspring (e.g., maternal exposure prior to conception or postnatally or similar levels of exposure of other individuals [partner exposure]).^67^ Negative control designs alert the analyst to uncontrolled confounding because if any of the effect estimates among the negative controls are positive, the effect measure of interest is likely confounded. Putting this design to practice, researchers using paternal exposure during pregnancy as a negative control in the Avon Longitudinal Study of Parents and Children found no evidence that maternal alcohol and tobacco consumption during pregnancy were more strongly associated with childhood IQ than paternal alcohol and tobacco consumption.^72^ In a second negative control study using ALSPAC data, researchers found that offspring of mothers who consumed any alcohol at 18 weeks of gestation had a 17% increased risk of having a diagnosis of depression at age 18.^73^ There was no clear evidence of association between partners’ alcohol consumption at 18 weeks of gestation and increased risk of offspring depression. The investigators concluded that the negative control comparison of paternal drinking provided some evidence that the association between PAE and depression at age 18 may be causal and warranted further investigation and replication.^73^ Although one must critically evaluate the potential for causality by the selected negative control (e.g., epigenetic effects in the case of paternal exposure), if it is deemed that there is no plausible mechanism by which the negative control could affect the outcome, the types of analyses should be conducted and reported.

## Conclusions

### Limitations of This Review

When reviewing the findings presented here, several limitations should be considered. First, restricting findings to published research introduces the potential for publication bias. However, given that many studies reviewed here yielded null findings, it is unlikely that publication bias greatly affects the results. Second, the high degree of heterogeneity in methods and the exposure definition prevented a meta-analysis.

Third, and most importantly, although three databases were searched, this review is a narrative review only and should not be interpreted as a systematic review. The search process used search terms “low, light, mild, or moderate,” and the authors then looked for predefined criteria for the categorization. Accordingly, there could be additional studies that included similar exposure ranges but did not use those terms; other studies may have included a population with a mean PAE that fell within a range described as low or moderate, but the study did not actually set minimum or maximum parameters for defining the exposure. Either scenario would have resulted in exclusion of those studies from this review. For example, Parry et al. examined several categories of alcohol exposure (i.e., 0 g/week, > 0–29 g/week, 30–59 g/week, 60–89 g/week, 90–119 g/week, and > 120 g/week)^74^ that overlap with definitions of low/light or moderate PAE in many of the studies included here. However, the study was excluded because the authors did not label these categories as low, moderate, or high exposure. Similarly, Beauchamp et al. enrolled a birth cohort with a PAE group that had a median of 0.84 ounces absolute alcohol per day during the periconceptual period and 0.3 ounces absolute alcohol per day during pregnancy.^75^ Although this sample may have overlapped with other samples in this review of moderate PAE, it likely also included infants who would have been categorized as low PAE and some who would have been categorized as high PAE, given that no minimum or maximum criteria were set for inclusion. Therefore, the study was excluded from this review. Attempting to include all such studies would require (1) searching for any study that had any measure of PAE, and (2) having clearly defined and accepted standards for categorizing low and moderate PAE. Such manual abstraction and classification in the absence of consensus definitions were beyond the feasibility of this review.

Fourth, the studies reviewed here assessed a variety of neurodevelopmental outcomes that may differ in sensitivity to alcohol based on the measure or the timing of administration. It was beyond the scope of this review to comment on each measure’s sensitivity and psychometric properties, although both also may contribute to the heterogeneity in findings. Finally, this review was limited to neurodevelopmental outcomes. There are other health and behavioral outcomes potentially affected by PAE that manifest across the life course and also warrant systematic investigation.

### Public Health and Clinical Implications

Although pregnant women may want to know whether there is a safe drinking threshold in pregnancy, this question is difficult to answer in human research. As seen in this review, findings are heterogeneous across studies, and many methodological limitations impair ability to validly estimate the potential consequences of low to moderate PAE. Moreover, the public should not confuse inconsistent evidence and insignificant findings to indicate absence of an effect. Accordingly, the only way to be certain to avoid adverse outcomes associated with alcohol exposure is to follow current guidelines to abstain from alcohol during pregnancy. Given that many women do not plan pregnancy, those who have had alcohol exposure prior to learning they are pregnant should avoid continued use.

### Summary and Future Directions

Although this review generally found null associations between low to moderate PAE and adverse neurodevelopment, the issue is far from resolved. There is no consensus in the literature on the level of harm that low to moderate prenatal alcohol exposure may cause, and the differential vulnerability resulting from influences such as concurrent exposures to other substances, genetics, and other factors may prevent a clear conclusion. Although the evidence base continues to expand, substantial methodological limitations impair synthesis of study findings. Use of alternative study designs may help advance research of the effects of low to moderate PAE on adverse outcomes, and the authors look forward to the expansion of these methodologies in the field. In addition, expanding reviews to capture other outcomes, including physical and behavioral outcomes, as they emerge across the life course is of great interest.

## Figures and Tables

**Appendix 1:** Studies of Low to Moderate Prenatal Alcohol Exposure and Neurodevelopmental Outcomes

Author (Year)	Data Source (*n*)	Setting; Offspring Birth Years	Dose of PAE: Low/Light	Dose of PAE: Moderate	Definition of One Drink/Unit of Alcohol	PAE Measurement	Outcome; Offspring Age at Measurement	Covariates	Finding
Barbuscia et al. (2019)^39^	Millennium Cohort Study (*n =* 10,454)	United Kingdom 2000–2002	< 1–2 units per week per occasion	< 3–6 units per week or < 3–5 units per occasion	Half-pint of beer, glass of wine, or single measure of spirits or liquor	Interview at 9 months postpartum	British Ability Scale (BAS); ages 3, 7, and 11 Bracken School Readiness Assessment; age 5 Strengths and Difficulties Questionnaire (SDQ); ages 3, 5, 7, 11, and 14	Child gender, mother’s ethnic background, low birth weight, maternal health, sociodemographic characteristics, and maternal cognitive ability	Light PAE was not associated with cognitive ability, internalizing, or externalizing behavioral problems. Moderate PAE was associated with better cognitive ability on 1 of 9 measures (others null) and worse externalizing problems at age 11 (null at other ages).
Bay et al. (2012)^27^	Danish National Birth Cohort Lifestyle During Pregnancy Study (*n =* 685)	Denmark 1997–2003	Low to moderate: weekly alcohol intake 1 to 14 drinks per week Analyzed as categories: 1 to 4 drinks per week 5 to 8 drinks per week 9 to 14 drinks per week		12 g pure alcohol	Self-reported at 17 weeks of gestation	Movement Assessment Battery for Children; age 5	Paternal education, maternal IQ, prenatal maternal smoking, maternal age, parity, maternal binge drinking episodes during pregnancy, prenatal and postnatal marital status, postnatal parental smoking, maternal pre-pregnancy body mass index (BMI), child sex, age at testing, health status, hearing and vision on day of testing, family/home environment, physical activity	No association existed between low to moderate PAE and total motor impairment or any motor skill subscales.
Chen (2012)^48^	National Longitudinal Survey of Youth (*n =* 1,618)	United States 1986–2000	Light to moderate: < 3 or 4 days a month		Not reported	Maternal report postpartum; < 23 months	Modified Rothbart Infant Behavior Questionnaire; ages < 23 months	Sibling fixed effects model with control for prenatal smoking, poverty status, marital status during pregnancy, prenatal care in first trimester (T1), parity, child sex	Light to moderate PAE was associated with greater difficultness of infant. No associations were found with positive mood of fearfulness subscales.
Cluver et al. (2019)^35^	Safe Passage Study (*n =* 500)	South Africa 2007–2015	Mild to moderate: ≤ 3 drinks in one sitting; never binge		14 g ethanol	Modified alcohol timeline follow-back around conception, up to 4 times during pregnancy, and at 1 month postpartum	Kaufman Assessment Battery for Children (KABC-II), a developmental neuropsychological assessment (NEPSY-II), Preschool Child Behavior Checklist (CBCL); age 4	Maternal age, education, smoking, marijuana use, and methamphetamine use	No associations existed with neurodevelopmental or behavioral outcomes.
Falgreen Eriksen et al. (2012)^29^	Danish National Birth Cohort Lifestyle During Pregnancy Study (*n =* 1,628)	Denmark 1997–2003	1 to 4 drinks per week	5 to 8 drinks per week	12 g pure alcohol	Self-reported at 17 weeks of gestation	Wechsler Preschool and Primary Scale of Intelligence—Revised; age 5	Parental education, maternal IQ, maternal smoking in pregnancy, child’s age at testing, child’s gender, parity, maternal marital status, maternal age and BMI, maternal binge drinking in pregnancy, family/home environment, prenatal/postnatal smoking, child’s health status, hearing and vision abilities	No association existed between low or moderate PAE and IQ.
Forrest et al. (1991)^42^	Dundee antenatal clinics (*n =* 592)	Scotland 1985–1986	Mild: 1–49 g per week	Moderate: 50–99 g per week	10 g absolute alcohol (AA) (equivalent to one glass wine, half-pint beer, or one standard measure spirits)	Interviewer-administered questionnaire; T1, second trimester (T2)	Bayley Scales of Infant Development (BSID) mental development index (MDI) and psychomotor development index (PDI); 18 months	Maternal cigarette consumption, age, and social class; child’s sex, birth weight, and gestational age	Mild PAE in early pregnancy was not associated with MDI or PDI. Moderate PAE in early pregnancy was not associated with MDI or PDI.
Gallagher et al. (2018)^50^	Millennium Cohort Study (*n =* 12,595)	United Kingdom 2000–2002	≤ 1–2 units per week or at any one time in pregnancy	≤ 3–6 units per week or 3–5 units at any one time in pregnancy	Half-pint beer, glass wine, or single measure spirits or liquor	Interview at 9 months postpartum	Maternal report of autism spectrum disorder (ASD) diagnosis; age 11	Parental age, household income, maternal education, social deprivation, ethnicity, marital status, maternal smoking, BMI, hypertension, diabetes, depression treatment, maternal smoking	No association existed between light or moderate PAE and ASD.
Goldschmidt et al. (2004)^49^	Maternal Health Practices and Child Development Study (*n =* 608)	United States 1984–1987	Light/moderate: < 1 drink per day		Not reported	Maternal interview at gestation months 4 and 7, and postnatally	Wide Range Achievement Test–Revised and Peabody Individual Achievement Test–Revised; age 10	Sociodemographic status, child characteristics and environment, maternal psychosocial status, current maternal substance use, and prenatal tobacco and illicit drugs other than marijuana	No association existed between light to moderate PAE and academic performance.
Halliday et al. (2017)^17^	Asking Questions About Alcohol in Pregnancy (*n =* 554)	Australia 2011–2012	≤ 20 g AA per occasion and ≤ 70 g AA per week	21–49 g AA per occasion and ≤ 70 g AA per week	10 g alcohol	Self-reported questionnaire at 13 weeks, 26 weeks, and postnatal interview; analyzed as T1, T2, and third trimester (T3)	BSID, Brief Infant-Toddler Social and Emotional Assessment; Infant/Toddler Sensory Profile; age 2	Maternal age, maternal education, household income, ethnicity, language spoken in home, pre-pregnancy BMI, folic acid supplementation, smoking in pregnancy, age started drinking regularly, paternal drinking	Low to moderate PAE was not associated with most neurodevelopmental outcomes. Low PAE in T1 with abstinence in T2 and T3 was associated with more sensation seeking.
Hutchinson et al. (2019)^20^,^52^	Triple B Pregnancy Cohort Study (*n =* 1,324)	Australia 2009–2013	≤ 7 drinks per week and ≤ 2 drinks per occasion	≤ 7 drinks per week and > 2 to ≤ 4 drinks per occasion	10 g alcohol	Interview during T1, T2, and at 8 weeks postpartum; analyzed as T1, T2, and T3	BSID-III gross motor development, age 12 months	Age at birth, education, Socio-Economic Indexes for Areas, state of residence, country of birth, single-parent household, Aboriginal and Torres Strait Islander status, native language, substance use, pregnancy anxiety, IQ, parity, BMI, gestational age	No association existed between low or moderate PAE and gross motor development.
Ichikawa et al. (2018)^34^	Japanese Study of Stratification, Health, Income and Neighborhood (*n =* 1,600)	Japan 2010–2013	Drinking rarely to 1 to 4 times per month	Not applicable (N/A)	Not reported	Self-reported at offspring age 2–18 (same age as outcome measurement)	CBCL; mean age 9 (*SD* 4.4, age range 2–18)	Sibling analysis and adjustment for child’s age, sex, parent's age, education, working status, family income, prenatal smoking, domestic violence, parental drinking	Low PAE was associated with greater anxiety problems, internalizing problems, and overall problems. Low PAE was not associated with several other CBCL-measured outcomes (e.g., externalizing behavior).
Kelly et al. (2009)^36^	Millennium Cohort Study (*n =* 9,460)	United Kingdom 2000–2002	≤ 1–2 units per week or per occasion	≤ 3–6 units per week or 3–5 units per occasion	Half a pint of beer, a glass of wine, or a single measure of spirits or liquor	Interview at 9 months postpartum	SDQ, BAS; age 3	Child’s age, birth weight, mother’s age at the time of birth, number of children in the household, mother’s education, mother’s drinking, mother’s smoking habits, household income, pregnancy planned, mother’s occupational class, mother’s Kessler Psychological Distress Scale (K6) score, warmth of relationship between mother and child, parental discipline	Light PAE was not associated with behavioral or cognitive problems. In boys, light drinking was associated with lower conduct and hyperactivity problems, and higher cognitive ability. Moderate drinking was not associated with any outcome in either girls or boys.
Kelly et al. (2012)^37^	Millennium Cohort Study (*n =* 11,513)	United Kingdom 2000–2002	≤ 1–2 units per week or per occasion	≤ 3–6 units per week or 3–5 units per occasion	Half-pint of beer, glass of wine, or single measure of spirits or liquor	Interview at 9 months postpartum	SDQ, BAS; age 5	Child’s age, birth weight, mother’s age at time of birth, number of children in the household, mother smoked during pregnancy, pregnancy planned, parental income, highest parental educational qualification, highest parental occupation, mother’s K6 score, parental discipline, child made to follow instructions, mother’s parental competence, closeness of relationship between mother and child, mother’s current drinking	No associations existed between light or moderate PAE and behavioral problems in girls or boys. Light PAE was associated with better cognitive ability scores in boys, null in girls; moderate PAE was not associated with cognitive ability scores.
Kelly et al. (2013)^38^	Millennium Cohort Study (*n =* 10,285)	United Kingdom 2000–2002	1–2 units per week or per occasion during pregnancy	N/A	Half-pint beer, glass of wine, or single measure of spirits or liquor	Interview at 9 months postpartum	SDQ, BAS; age 7	Mother’s age, planned pregnancy, maternal smoking, parity, ethnicity, lone-parent family, life satisfaction, relationship quality, social networks, number of children in household, child’s age, highest parental educational qualification, parental income, mother’s mental health, parental discipline strategies, mother’s self-rated competence, mother’s closeness with child, mother’s current drinking	No association existed between light PAE in pregnancy and behavioral or cognitive development.
Kesmodel et al. (2012)^26^	Danish National Birth Cohort Lifestyle During Pregnancy Study (*n =* 1,628)	Denmark 1997–2003	1 to 4 drinks per week	5 to 8 drinks per week	12 g pure alcohol	Interview at 17 weeks	Behavior Rating Inventory of Executive Function, Wechsler Preschool and Primary Scale of Intelligence—Revised, Test of Everyday Attention for Children at Five; age 5	Parental education, maternal IQ, prenatal maternal smoking, child’s gender, child's age at testing, test administrator, parity, maternal marital status, maternal age, maternal BMI, prenatal maternal average number of drinks per week, home environment, postnatal parental smoking, health status, hearing and vision abilities	No association existed between low or moderate PAE and executive function, intelligence, or attention.
Kilburn et al. (2015)^32^	Danish National Birth Cohort Lifestyle During Pregnancy Study (*n =* 1,333)	Denmark 1997–2003	1–4 drinks per week	5–8 drinks per week	12 g	Interview at 17 weeks	Sternberg paradigm to assess information processing time and choice reaction time; age 5	Parity, prenatal maternal smoking, maternal pre-pregnancy BMI, length of parental education, marital status, postnatal parental smoking, child health status, family/home environment index, breakfast irregularity, maternal depression, parental alcohol use, hearing ability, and vision ability	No association existed between maternal drinking and choice reaction time or information processing time.
Larkby et al. (2011)^47^	Clinical sample at maternity clinic (*n =* 592)	United States 1989–1991	Average drinks per day in T1 ≤ 0.4	Average drinks per day T1 > 0.4 to < 0.89	Not reported	Maternal report at 4th and 7th gestational months and at delivery	Computerized Diagnostic Interview Schedule-IV to measure conduct disorder; age 16	Prenatal exposure to tobacco, marijuana, cocaine, and other illicit drugs; income; race; gender; parenting style; life events; home environment; family history of alcohol problems; and maternal lifetime psychopathology	No association existed between light or moderate PAE and conduct disorder in adolescents.
Lees et al. (2020)^46^	Adolescent Brain Cognitive Development Study (*n =* 9,719)	United States 2005–2008	2.3 drinks per week (1st 7 weeks of pregnancy) 1.1 drinks per week through gestation	N/A	Not reported	Maternal report at offspring age 9–10 (same time as outcome measurement)	(1) CBCL, (2) Schedule for Affective Disorders and Schizophrenia for School-Age Children (K-SADS), (3) Impulsive Behavior Scale for Children–Short Form, (4) Behavioral Avoidance and Behavioral Inhibition Scales, (5) Cash Choice Task, (6) Rey Auditory Verbal Learning Test, (7) NIH Toolbox fluid intelligence battery, (8) brain imaging; ages 9–10	Birth weight, preterm birth, sex at birth, race/ethnicity, youth age at time of assessment and school grade performance, maternal age at birth, maternal depression, and other substance use during pregnancy (tobacco, cannabis, cocaine)	Light stable PAE and light PAE reduced in pregnancy were associated with greater behavioral and psychological problems (CBCL: internalizing, externalizing, attention problems, total; K-SADS: anxiety, specific phobias) and differences in cerebral and regional brain volume.
Maher et al. (2022)^23^	SCOPE (Screening of Pregnancy Endpoints) and BASELINE (Babies After SCOPE: Evaluating Impact on Neurological and Nutritional Endpoints) (*n =* 1,507)	New Zealand, Australia, Ireland, United Kingdom 2004–2011	1–7 units per week		8 g or 10 ml (1 dl) pure alcohol	Interview at 15 weeks of gestation	CBCL; ages 2 and 5 Kaufman Brief Intelligence Test; age 5	Maternal age, maternal education, marital status, family income, maternal BMI, maternal smoking status at 15 weeks of gestation, and infant sex	No associations existed between low PAE and behavioral outcomes at age 2 or age 5.
McCormack et al. (2018)^18^	The Triple B Study (*n =* 1,331)	Australia 2008–2013	≤ 7 drinks per week and ≤ 2 drinks per occasion	≤ 7 drinks per week and > 2 to ≤ 4 drinks per occasion	10 g alcohol	Interview at T1, T2, and 9 weeks postpartum	BSID; age 12 months	Household SES, maternal age, maternal education level, Aboriginal or Torres Strait Islander origin, country of birth, single-parent household, first language, tobacco use, illicit substance use, anxiety, IQ, parity, and BMI	Low levels of PAE in T2 and T3 were associated with slightly higher cognitive scores. No associations existed with low or moderate PAE in T1. Moderate PAE in T2 and T3 was not analyzed.
Mitchell et al. (2020)^41^	Millennium Cohort Study (*n =* 13,004)	United Kingdom 2000–2002	≤ 3 to 7 drinks per week	≤ 8 to 14 drinks per week	Half a pint of beer, a glass of wine, or a single measure of spirits or liquor	Interview at 9 months postpartum	Parental report of attention-deficit/hyperactivity disorder (ADHD) diagnosis, SDQ; age 7	Gender, gestational age at delivery, parity, paternal age, maternal age, maternal smoking status, maternal pre-pregnancy BMI, household income, maternal education, ethnicity, and marital status	Neither light nor moderate drinking were associated with ADHD, abnormal SDQ scores, or hyperactivity scores.
Niclasen et al. (2014)^25^	Danish National Birth Cohort (*n =* 37,152)	Denmark 1996–2002	Cumulative > 0 to 5 drinks per pregnancy; average > 0 to 2 drinks per week	Cumulative > 5 to 90 drinks per pregnancy; average 2 to 4 drinks per week	12 g pure alcohol	Maternal report at gestational week 16, week 30, and at 6 months postpartum	SDQ; age 7	Paternal smoking, parental education, parental pre-pregnancy psychiatric diagnoses, and maternal psychological well-being in pregnancy	No associations existed between light or moderate PAE with parent-rated conduct, emotional, hyperactivity/inattention, or peer problems. Small protective associations existed between low and moderate PAE in early part of pregnancy with internalizing problems among boys (not girls).
O’Callaghan et al. (2007)^22^	Mater-University of Queensland Study of Pregnancy (*n =* 5,139)	Australia 1981–1984	Low: < 0.5 glass per day 0.5 to 1 glass per day		0.5 oz AA	Reported at first prenatal clinic visit and after delivery	CBCL; age 14	Cigarette smoking in early and late pregnancy, maternal BMI < 18.5, social risk score (low maternal education, maternal age < 19, single-parent status, or low income in pregnancy or at age 14)	No associations existed between light or moderate alcohol, neither early nor late in gestation, and attention, learning, or cognitive outcomes.
O’Leary et al. (2009)^19^	Western Australian Survey of Health (*n =* 1,739)	Australia 1995–1996	≤ 20 g alcohol per occasion, with a frequency of less than weekly up to 6 days per week	10 g to < 50 g alcohol per occasion, with a frequency ranging from less than weekly up to daily	10 g alcohol	Questionnaire at 12 weeks postpartum	Ages & Stages Questionnaire Communication Scale; age 2	McMaster’s family functioning, parenting scale, partner present, maternal depression, anxiety, stress, maternal age at delivery, income, marital status, parity, education, smoking, use of tranquilizers, illicit drug use	No association existed between low PAE and language delay.
O’Leary et al. (2010)^15^	Western Australian Survey of Health (*n =* 2,224)	Australia 1995–1996	< 70 g alcohol per week and ≤ 10–20 g per occasion	≤ 70 g alcohol per week and between 21 g and 49 g per occasion	10 g alcohol	Questionnaire at 12 weeks postpartum	Ages & Stages Questionnaire (language delay); age 2 CBCL; ages 2, 5, and 8	Antenatal covariates (maternal age, marital status, parity, ethnicity, income, maternal smoking, and use of illicit drugs, tranquilizers, and sleeping tablets during pregnancy), postnatal covariates (marital status, income, treatment for postnatal depression, postnatal depression, family functioning, parenting style, tension in the family due to alcohol and maternal depression, anxiety, and stress	No association existed between low PAE and language delay (also reported in paper above). Moderate PAE in T1 was associated with increased odds of anxiety/depression but not with somatic or aggressive problems. No association existed between low PAE and any CBCL outcome in children ages 2, 5, or 8.
O’Leary et al. (2013)^21^	Western Australian Survey of Health (*n =* 4,056)	Australia 1995–1996	1–2 drinks per occasion and < 7 drinks per week	3–4 standard drinks per occasion and ≤ 7drinks per week	10 g alcohol	Questionnaire at 12 weeks postpartum	Western Australian Literacy Numeracy Assessment measures whether children met benchmarks for reading, writing, spelling, and numeracy; ages 8–9	Maternal age, education, marital status, ethnicity, parity, illicit and/or tranquilizer drug use, smoking, income, and languages spoken at home	Low PAE was associated with lower odds of missing numeracy academic benchmark; not statistically significant for reading, spelling, or writing benchmark. Moderate PAE was not associated with academic underachievement.
Robinson et al. (2010)^16^	Western Australian Pregnancy Cohort (*n =* 1,744)	Australia 1989–1991	Occasional: ≤ 1 drink per week Light: 2 to 6 drinks per week	7 to 10 drinks per week	10 g alcohol	Questionnaire at 18 and 34 weeks of gestation	CBCL; age 14	Maternal age, maternal education, presence of biological father in family home, family income, stress in pregnancy, maternal cigarette smoking, child’s age	Light and moderate PAE in first 3 months of pregnancy was associated with lower internalizing and externalizing problem scores; no associations were observed for late pregnancy PAE.
Rodriguez et al. (2009)^33^	Three cohorts from Nordic Network on ADHD (*n =* 21,678)	Denmark, Finland 1984–2002	1 to 4 drinks per week	N/A	Not reported	Maternal report of average weekly exposure at ~16–32 weeks of gestation	SDQ, Rutter Scale; ages 7–15	Smoking, social adversity, birth weight, gestational age (analyzed in three cohorts to analyze differences in participant characteristics)	Low PAE was not associated with inattention/hyperactivity.
Sayal et al. (2007)^43^	Avon Longitudinal Study of Parents and Children (*n =* 9,086)	United Kingdom 1991–1992	< 1 glass per week	N/A	8 g alcohol (equivalent to 1 glass)	Self-reported questionnaire at 18 weeks of gestation	Parental SDQ; 47 and 81 months Teacher SDQ; 92 to 108 months	Smoking, cannabis use, and use of illicit drugs in T1, highest level of maternal education, home ownership, marital status, parity, maternal age group, high Edinburgh Postnatal Depression Scale score, child ethnicity, gestational age group, and birth weight	Low PAE was associated with worse parental and teacher-rated SDQ scores among girls, but not among boys.
Sayal et al. (2013)^40^	Avon Longitudinal Study of Parents and Children (*n =* 10,558)	United Kingdom 1991–1992	< 1 glass per week	N/A	8 g alcohol (equivalent to 1 glass)	Self-reported questionnaire at 18 weeks of gestation	SDQ, Kay Stage 2 school examinations; age 11	Maternal age, parity, highest level of maternal education, daily frequency of smoking, use of cannabis and/or other illicit drugs during T1, home ownership, whether currently married, maternal mental health, child’s gestational age, birth weight, and gender	No association existed between light PAE and outcomes. In girls, a suggestion of slightly worse outcomes appeared on parent-rated total SDQ score in those exposed to light PAE. Light PAE was not associated with Kay Stage 2 scores.
Singer et al. (2017)^44^	Study to Explore Early Development Case-Control Study (*n =* 2,515)	United States 2003–2006	Light: < 1 drink per week; 1–2 drinks per week		1 beer, 1 glass wine, 1 mixed drink, or 1 shot liquor	Maternal report* at 55 months postpartum (range: 29–68 months) *same time as outcome measurement	ASD diagnosis measured with the Autism Diagnostic Observation Schedule (child report) and the Autism Diagnostic Interview Revised (caregiver report); ages 30–68 months	Child’s sex, total household income in the year prior to the pregnancy, self-reported maternal race/ethnicity, maternal education at delivery, maternal parity, at least one maternal psychiatric condition, maternal smoking in any month during preconception and pregnancy, and maternal age at birth	< 1 drink per week in month 1 or month 2 was not associated with ASD; 1–2 drinks in month 1 were associated with lower odds of ASD (1–2 drinks in month 2 not significant).
Skogerbø et al. (2012)^31^	Danish National Birth Cohort Lifestyle During Pregnancy Study (*n =* 1,628)	Denmark 1997–2003	1 to 4 drinks per week	5 to 8 drinks per week	12 g pure alcohol	Interview at 17 weeks	Behavior Rating Inventory of Executive Function parent and teacher forms; age 5	Parental education, maternal IQ, prenatal maternal smoking, child’s age at testing, child’s gender, maternal binge drinking, maternal age, parity, maternal marital status, family home environment, postnatal parental smoking, pre-pregnancy maternal BMI, health status of child	No association existed between low or moderate PAE and executive function.
Skogerbø et al. (2013)^30^	Danish National Birth Cohort Lifestyle During Pregnancy Study (*n =* 1,628)	Denmark 1996–2002	1 to 4 drinks per week	N/A	12 g pure alcohol	Interview at 17 weeks	Parent- and teacher-rated SDQ, age 5	Maternal binge drinking, parental education, maternal IQ, prenatal maternal smoking, child’s age at testing, child’s gender, maternal age, parity, maternal marital status, family-home environment, postnatal parental smoking, pre-pregnancy maternal BMI, and child’s health status	No association existed between low to moderate alcohol consumption and offspring behavior.
Underbjerg et al. (2012)^28^	Danish National Birth Cohort Lifestyle During Pregnancy Study (*n =* 1,628)	Denmark 1997–2003	1 to 4 drinks per week	5 to 8 drinks per week	12 g pure alcohol	Interview at 17 weeks	Test of Everyday Attention for Children at Five, age 5	Parental education, maternal IQ, maternal smoking in pregnancy, child’s age at testing, gender, and tester were considered core confounding factors, whereas the full model also controlled the following potential confounding factors: maternal binge drinking or low to moderate alcohol consumption, age, BMI, parity, home environment, postnatal smoking in the home, child’s health status, and indicators for hearing and vision impairments	No association existed between low or moderate PAE and attention.
Weile et al. (2020)^24^	Aarhus Birth Cohort (*n =* 48,072)	Denmark 1998–2012	Analyzed as categories: < 1, 1, 2, or ≥ 3 drinks per week	N/A	12 g pure alcohol	Questionnaire in early pregnancy (median 11 weeks)	ADHD diagnosis from Danish health registries, median age 12 (up to age 19)	Maternal age, highest attained educational level, chronic disease, presentational BMI, smoking in pregnancy, parity, birth year, binge drinking	Up to 1 drink per week PAE was associated with lower risk of ADHD; no associations existed with 2 or more drinks per week.
Williams Brown et al. (2010)^45^	Early Childhood Longitudinal Studies— Birth Cohort (*n =* 10,700)	United States 2001	Low to moderate: 0 to 4 drinks per week Analyzed in categories < 1 drink per week, 1–3 drinks per week		Not reported	Maternal report at 9 months postpartum (range 6 to 22 months)	Bayley Short Form—Research Edition; Nursing Child Assessment Teaching Scale, Behavior Rating Scale, Infant/Toddler Symptom Checklist; age 9 months	Race, poverty, child’s age at assessment	< 1 drink or 1 to 3 drinks were not associated with most outcomes including Bayley Mental or Motor subscales, sensory regulation variables, and behavior rating scale. < 1 drink and 1 to 3 drinks were associated with undesirable social engagement and child interaction.

*Note:* AA, absolute alcohol; ADHD, attention-deficit/hyperactivity disorder; ASD, autism spectrum disorder; BAS, British Ability Scale; BMI, body mass index; BSID, Bayley Scales of Infant Development; BSID-III, Bayley Scales of Infant and Toddler Development, third edition; CBCL, Child Behavior Checklist; dl, deciliter; g, grams; K6 score, Kessler Psychological Distress Scale score; K-SADS, Schedule for Affective Disorders and Schizophrenia for School-Age Children; MDI, mental development index; ml, milliliter; N/A, not applicable; NEPSY-II, NEuroPSYchological assessment, second edition; NIH, National Institutes of Health; PAE, prenatal alcohol exposure; PDI, psychomotor development index; *SD*, standard deviation; SDQ, Strength and Difficulties Questionnaire; SES, socioeconomic status; T1, first trimester; T2, second trimester; T3, third trimester.
